# Bottlenecks to child health service delivery – how can Health System Performance Assessment help?

**DOI:** 10.1093/eurpub/ckac129.332

**Published:** 2022-10-25

**Authors:** N Shuftan, D Rajan

**Affiliations:** 1 Department of Healthcare Management, Technische Universitaet Berlin, Berlin, Germany; 2 Brussels Hub, European Observatory on Health Systems and Policies, Brussels, Belgium; 3 WHO, Geneva, Switzerland

## Abstract

Health System Performance Assessment (HSPA) is an established tool that can help identify issues with the way health systems work to achieve their intended goals, improve transparency and account-ability, and provide stimulus for reform. However, HSPA frameworks tend to be adult-centric and therefore not generally sensitive to child health challenges. Using a new HSPA framework for Universal Health Coverage, we show how child health tracer conditions can be used to pinpoint system under-performance and flag major bottlenecks impeding service delivery overall. Based on the HSPA framework, health system bottlenecks can be traced backwards to explore possible origins (areas to be targeted for improvement) or forwards to understand potential implications for the achievement of health system goals. The following qualitative and quantitative data are used to highlight major bottlenecks to child health service delivery: eight health system-focused child health assessments which show that one of the main bottlenecks is weak primary care coverage of sexual, reproductive, maternal, newborn, child, and adolescent health services, leading to parents bypassing PHC in favour of hospitals; results from WHO missions (e.g. in Romania and Tajikistan) showing that paediatric patients were often admitted to hospitals for treatment, even for conditions that could be safely managed in the outpatient setting; quantitative data on responsiveness, user experience, client satisfaction and people-centredness from publicly available datasets and national datasets obtained in collaboration with WHO Country Offices for national level data, to contextualize the aforementioned findings.

